# Parapapillary choroidal dense pigmentation in young healthy eyes: a cross-sectional study

**DOI:** 10.1186/s40942-026-00865-8

**Published:** 2026-05-08

**Authors:** Yuki Uto, Takehiro Yamashita, Kazuki Fujiwara, Aiko Iwase, Hiroto Terasaki, Kumiko Nakao, Taiji Sakamoto

**Affiliations:** 1 Department of Ophthalmology, Kagoshima University Graduate School of Medical and Dental Sciences, Kagoshima, Japan; 2 Tajimi Iwase Eye Clinic, Gifu, Japan

**Keywords:** Parapapillary choroidal dense pigmentation, Pigmented conus, Axial length

## Abstract

**Background:**

While observing fundus photographs from exploratory research investigating individual differences in the fundus, we discovered a black band around the optic nerve head that had not been reported in previous studies. Such eyes with parapapillary choroidal dense pigmentation (PCP) have a pigmented conus visible on color fundus photography. This study aimed to determine the prevalence of PCP in young healthy eyes and examine its relationships with axial length, optic disc tilt, and conus area.

**Methods:**

This prospective, observational, cross-sectional study included the right eyes of 133 participants, who were examined between November 1, 2010 and February 20, 2012. Among them, 117 right eyes of 117 patients were finally analyzed. Participants underwent comprehensive ophthalmologic examinations, including axial length measurement, fundus photography, and optic disc optical coherence tomography (OCT). Based on their color fundus photographs and optic disc cross-sectional OCT images, eyes were categorized into the non-PCP, temporal-PCP, and circum-PCP groups. Optic disc tilt was evaluated using a sine curve based on the retinal nerve fiber layer B-scan images. The conus area in the color fundus images was calculated using ImageJ and corrected using Bennett’s formula. The Steel–Dwass test was used to perform multiple comparisons of the axial length, optic disc tilt, and conus area among the three groups.

**Results:**

The mean age and axial length of the participants were 25.8 years and 25.5 mm, respectively. Of the 117 eyes, 49, 17, and 51 had non-PCP, temporal-PCP, and circum-PCP, respectively. The axial length (*p* = 0.011) and conus area (*p* = 0.047) were significantly shorter and smaller, respectively, for the circum-PCP group than for the non-PCP group. No significant differences were observed in the other intergroup comparisons.

**Conclusions:**

OCT findings revealed that PCP appears black due to choroidal pigment. The eyes with circumferential PCP had shorter axial lengths and smaller conus areas than that of those without, suggesting that it may occur more likely in eyes with less axial elongation.

## Background

Hyper- and hypopigmentation around the optic nerve head was reported by Jonas et al. in 1988, and these were named parapapillary atrophy alpha (PPA alpha) [1, 2]. Irregular pigmentation, PPA alpha, was subsequently reported to be associated with relative scotoma [3] and more extensively in eyes with early glaucoma and ocular hypertension than in normal eyes [4, 5]. However, its frequency and size did not differ between the eyes with normal tension glaucoma and normal eyes [6]. A study reported that PPA alpha was detected in all 47 eyes with open-angle glaucoma, and its area was significantly correlated with the mean deviation of standard automated perimetry [7].

Histological examination of human eyes has revealed that PPA alpha is associated with thickening and thinning of the retinal pigment epithelium (RPE) [8]. Dichtl et al. reported that PPA alpha is located outside PPA beta and in a specific part of the RPE layer [9]. Furthermore, a long-term observational study in rhesus monkeys showed that the PPA beta increased in size; however, the PPA alpha remained unchanged in size and frequency in a long-term observational study of rhesus monkeys [10].

A cross-sectional population study reported that the prevalence of zone alpha decreased by 0.4% per 10 years of age [11]. Jonas et al. reported an increase of approximately 0.6% in the PPA alpha area based on the findings of the Beijing study, which involved a 5-year follow-up. This increase was associated with older age and the coexistence of zone beta [12].

**Fig. 1:**
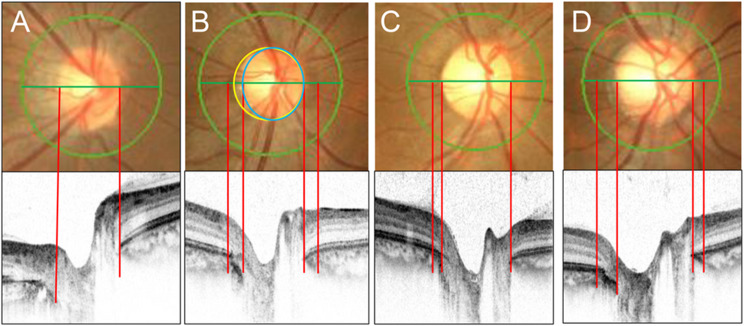
Individual variation of the parapapillary choroidal dense pigmentation (PCP). The eyes without PCP have a high density of choroidal vessels and a low density of choroidal stroma (**a**). The eyes with circumferential PCP have a low density of choroidal vessels and a high density of choroidal stroma (**b**). Eye with temporal-PCP (**c**). Eye with circumferential PCP and black conus (**d**)

We have been studying individual differences in the fundus of healthy young adults [13–15]. In this study, we observed a ring-shaped pigmented band around the optic disc that was distinct from PPA alpha, but continuous with PPA alpha, as shown in Fig. 1b. It appeared as black myopic conus in some eyes. Some eyes had a pigmented band around the entire optic disc, whereas others had it only on the temporal side (Fig. 1c).

On horizontal cross-sectional optical coherence tomography (OCT) image, there were no IS/OS line and thin RPE layer in the black myopic conus on the temporal side. Moreover, the IS/OS line was thin and the RPE layer was not thick in the black area on the nasal side (Fig. 1d). This suggested the black area observed on the fundus photograph to be attributable to a cause other than PPA alpha, which involves thickening of the RPE. The choroidal pigments were the only parts of the fundus structure that appeared black, other than the RPE. Therefore, we named this pigment band observed in the fundus as the parapapillary choroidal dense pigmentation (PCP).

This study investigated the relationships between the axial lengths, optic disc tilts, and conus areas of eyes with non-PCP, temporal-PCP, and circumferential-PCP.

## Methods

### Study design and study population

This prospective, cross-sectional, observational study enrolled 133 participants. Only right eyes were included. This was an exploratory study designed to investigate individual differences in the fundus. Examinations were performed from November 2010 through February 2012. Individuals with no history of ocular disease, based on a review of medical records and comprehensive ophthalmic examinations, were enrolled. The eligibility criteria were: 20–40 years of age; healthy eyes as determined by slit-lamp biomicroscopy, ophthalmoscopy, and OCT; best-corrected visual acuity of ≥ 0.1 logarithm of the minimum angle of resolution units; and intraocular pressure of ≤ 21 mmHg. The exclusion criteria were: eyes with ocular diseases such as glaucoma, pathologic myopia, and optic disc anomaly; known systemic diseases such as hypertension and diabetes; visual field defects; prior refractive or intraocular surgery; and poor OCT or fundus image quality. Eyes were excluded for the following reasons: glaucoma (*n* = 1), superior segmental optic disc hypoplasia (*n* = 3), and a history of refractive surgery (*n* = 3). An additional nine eyes were omitted because fundus parameters could not be reliably evaluated. Finally, data from 117 eyes (80 male and 37 female) were analyzed.

### Measurements

Each participant underwent a standardized set of ophthalmic examinations. These comprised slit-lamp biomicroscopy, fundus evaluation, intraocular pressure assessment with a pneumotonometer (CT-80; Topcon, Tokyo, Japan), and ultrasonic measurement of axial length (AL-2000; TOMEY, Nagoya, Japan). Objective refraction was obtained using an autorefractometer/keratometer (KR-8800; Topcon), and spherical equivalent values were used for analysis.

Color fundus photographs were obtained using the TRC-50LX (TOPCON, Tokyo, Japan). Color fundus photographs and the RNFL 3.4 mm circle scans were obtained using the Topcon 3D OCT-1000 Mark II (TOPCON, Tokyo, Japan). A Spectralis OCT device (Heidelberg Engineering, Germany) was used to obtain scanning laser ophthalmoscopy (SLO) images and cross-sectional views of the optic disc. The scan pattern was centered on the optic nerve head and comprised 73 horizontal B-scans spanning 15 degrees. The averages of the data of seven B-scans were obtained and used. Built-in image registration and eye-tracking functions were applied to minimize motion artifacts during image acquisition. Image quality was assessed by the operator, with confirmation of appropriate SD-OCT B-scan positioning within the image frame, proper centering on the optic nerve head, and an adequate quality score (> 20). The images were reacquired when necessary. The color fundus ophthalmoscopic and SLO fundus images were aligned using PowerPoint software based on the vascular pattern. The Spectralis OCT software was used to process and optimize SLO images, color fundus photographs, and cross-sectional OCT scans.

### Assessment of the PCP, conus area, and optic disc tilt

PCP was assessed based on evaluation of color fundus photographs and cross-sectional images of the optic disc. The PCP was defined based on the presence of a dark black parapapillary circular pigment band on ophthalmoscopy and dense choroidal stroma with low choroidal vessel density on OCT. Eyes without these features were allocated to the non-PCP group (Fig. 1A). Those with these features on the temporal side of the optic disc were allocated to the temporal-PCP group (Fig. 1C), and those with these features on the circumference of the optic disc were allocated to the circum-PCP group (Fig. 1B, D). The PCP and conus were independently assessed by two raters (KF and TY). The third rater (HT) determined the type of PCP in cases of disagreement.

Optic disc tilt was quantified according to a method previously reported by our group [14]. The optic disc tilt was quantified using the sine curve method based on the RNFL 3.4-mm circle scan, B-scan images obtained using the Topcon 3D OCT-1000 Mark II (TOPCON, Tokyo, Japan). The RPE course was plotted manually on the B-scan images. The coordinates of each pixel were determined automatically using ImageJ software. The coordinate system of the B-scan images was transformed such that the wave center served as the new origin. The converted data were fitted to the following sine wave equation using the curve fitting program of ImageJ:

$$\:y=a\times\:\mathrm{sin}\:(b\times\:x-c)$$

The amplitude (a) of the sine curve was defined as the degree of optic disc tilt. The conus area was measured from the fundus photographs using ImageJ. Conus area was calculated by subtracting number of pixels in the yellow oval from the blue oval (Fig. 1B). The image size decreases with increasing axial length because the fundus is farther from the camera. The magnification effect of the axial length was adjusted using Bennett’s formula (3.12 × 0.01306 × 100%/mm) [15, 16].

### Statistical analyses

The intra-rater agreement for the PCP was determined using the weighted Cohen’s kappa coefficient. The Kruskal–Wallis one-way analysis of variance and Steel–Dwass multiple comparison tests were used to determine the significance of the differences in the axial length, conus area, and optic disc tilt among the groups. Statistical analyses were performed using R (version 3.0.2; The R Foundation for Statistical Computing, Vienna, Austria). Statistical significance was set at *p* < 0.05.

## Results

**Fig. 2:**
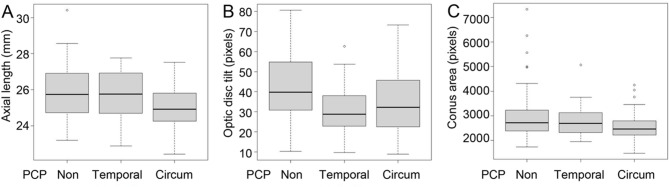
Box plot analysis of the differences between the groups. Differences in the axial length (**a**), optic disc tilt (**b**), and conus area (**c**) among the non-, temporal, and circumferential parapapillary choroidal dense pigmentation (PCP) groups are shown. The axial length and conus area were shorter and smaller, respectively, in the circum-PCP group than in the non-PCP group

The demographic information of the patients is provided in Table 1. The mean age and axial length of the patients were 25.8 years and 25.5 mm, respectively. The number of eyes without PCP, with temporal-PCP, and with circum-PCP were 49, 17, and 51, respectively. The inter-rater agreement for PCP was high (kappa = 0.81). The axial length of the circum-PCP group was significantly shorter than that of the non-PCP group (*p* = 0.011). However, the differences between the non-PCP and temporal-PCP groups (*p* = 0.914) and between the temporal-PCP and circum-PCP groups (*p* = 0.195) were not significant (Fig. 2A). The optic disc tilt did not significantly differ among the three groups (non vs. temporal, *p* = 0.061; temporal vs. circum, *p* = 0.729; non vs. circum, *p* = 0.108) (Fig. 2B). The conus area was significantly smaller for the circum-PCP group than for the non-PCP group (*p* = 0.047). However, the differences between the non-PCP and temporal-PCP groups (*p* = 0.908) and between the temporal-PCP and circum-PCP groups (*p* = 0.351) were not significant (Fig. 2C).

**Table 1:** Participant characteristics

	Mean ± standard deviation or Median (interquartile range)	Range
Age (years)	25.8 ± 4.0	22–40
Sex (male/female)	80 / 37	
Spherical equivalent (diopters)	-4.72 ± 3.33	-14.25-0.50
Axial length (mm)	25.45 ± 1.42	22.43–30.42
PCP (non/temporal/circum)	49 / 17 / 51	
Optic disc tilt (pixels)	37.69 ± 17.19	8.80–80.77
Conus area (pixels)	377.79 (0.00, 794.06)	0.00–4194.98

PCP: parapapillary choroidal dense pigmentation

## Discussion

To the best of our knowledge, this study presents the first characterization of PCP, a new morphological feature in young healthy eyes. We found a significant association between the circumferential PCP pattern and shorter axial length and smaller conus area. These results suggest that PCP may be a feature of less ocular elongation, distinguishing it from established myopic changes, and requires further investigation into its choroidal origin.

The axial length and conus area were shorter and smaller, respectively, for the circum-PCP group than in the non-PCP group. These results suggest that circum-PCP may be one of the hyperopic features. Myopic features [17] include myopic conus (PPA beta, gamma, and delta zones) [18], optic disc tilt [14], tessellation [19], vessel shift toward the fovea [13], and peripapillary nerve fiber elevation [15]. However, a few special hyperopic features have been reported in the fundus. Hyperopia is a refractive state of an eye with less eyeball elongation during growth [20, 21]. The choroid is very thick during infancy, but it gradually thins as the eyeball expands during growth [22]. This is easy to understand if you imagine a balloon inflating. For example, the area around the inlet of the green balloon is darker than the rest of the balloon. The morphology is similar when the optic disc is considered the inlet of the eyeball, with a ring-shaped area of concentrated choroidal pigment around the optic disc. A short axis and small conus suggest less bulging of the eyeball. The choroidal pigment remains dark around the disc in such eyes, creating a ring-shaped black area that may result in PCP.

The inlet of the balloon appears darker due to its weak inflation and thick wall. Which is the greater factor in PCP? The choroid in the PCP area appears to be rich in stromal components rather than thick, as observed in the OCT images (Figs. 1B–D). The choroidal vascular component is abundant up to the optic disc in eyes without PCP (such as those in Fig. 1a); however, the stromal component is scant. The proportions of the stromal components in the choroid vary greatly among individuals [22, 23]. The stromal and vascular components become thinner as axial length increases [23, 24]. This results in the visibility of the large choroidal vessels, which causes stronger tessellation and a redder fundus [25]. PPA alpha was present in 98.6% of the cases in an epidemiological study involving Indians [26]. This study focused on Japanese participants, whose fundus pigmentation is darker than that of Westerners. PCP may be present in eyes with dark fundus pigmentation and weak ocular dilation owing to individual and racial differences. Further research is needed to investigate the relationship between the ratio of choroidal stromal and vascular components [23] and PCP in various ethnic groups.

The axial length of the non-PCP group was longer than that of the circum-PCP group. This suggests that myopic fundus changes attenuate PCP. The conus area was larger for the group without PCP than for the group with PCP. Axial elongation causes the choroid to become paper-thin on the temporal side of the optic disc and the sclera becomes visible, resulting in a whitish myopic conus [18]. The nerve fibers on the nasal side of the optic disc are elevated and appear whitish in eyes with peripapillary nerve fiber elevation [15]. This may reduce the visibility of the choroid and the apparent blackness of the fundus. Optic disc tilt is also a myopic change, and we expected the circum-PCP group to have greater optic disc tilt than that of the non-PCP group. However, the difference was not significant. This suggests that optic disc tilt has less effect on PCP than does axial length and conus area. Further research is needed to investigate the relationship between myopic changes associated with axial elongation during growth and changes in PCP.

The PPA alpha reported by Jonas et al. [1, 2] may be influenced by RPE irregularity on the temporal side of the optic disc. However, the fundus findings in Fig. 1 show a black ring around the optic disc that is connected to PPA alpha. Therefore, RPE irregularity around the optic disc is unlikely. Moreover, the term PPA is inappropriate because PCP is not an atrophic change. PCP is a fundus finding derived from choroidal pigment and must be distinguished from the RPE irregularity.

This study has some limitations. First, it was a cross-sectional correlation study, and the chronological changes in PCP could not be observed. Second, it involved a young Japanese population. An epidemiological study showed that myopia was the most prevalent in the Japanese population [27]. PCP may be common in young healthy eyes, but the findings of this study may not be generalizable to other ethnic groups. In particular, as the amount of pigment in the fundus differs among races, research on PCP in non-Asian individuals is necessary. Since this study did not include emmetropia or hyperopia, future research should focus on PCP in populations with a wider distribution. Third, considering the significant improvements in OCT technology over the past decade, further research is needed to confirm PCP using the latest OCT technology. While the study introduces PCP as a novel morphological finding, its clinical implications remain unclear. It is necessary to investigate the state of PCP at birth, the process by which PCP changes during the growth period, and its relationship with disease.

## Conclusions

This study reports PCP, a fundus finding related to choroidal pigment around the optic disc. PCP may be observed in hyperopic eyes and may serve as an indicator of individual differences in fundus pigment. However, many aspects of PCP remain unclear, and further research is needed to explore ethnic differences, its relationship with quantified choroidal structure, and its association with ocular diseases.

## Data Availability

The datasets used and/or analyzed during the current study are available from the corresponding author on reasonable request.

## Abbreviations

OCT: Optical coherence tomography
PCP: Parapapillary choroidal dense pigmentation
PPA alpha: Parapapillary atrophy alpha
RPE: Retinal pigment epithelium
